# 

**Published:** 1897-01

**Authors:** 


### Communicated.

### REPORT OF THE COMMITTEE ON NATIONAL DENTAL MUSEUM AND LIBRARY.

*To the American Dental Association:*

The 35th Annual Meeting of the American Dental Association, by invitation and in line with precedents of other National associations of specialists, formally recognized the Army Medical Museum and Library as the National Museum and Library of the Dental Profession of the United States, and appointed the undersigned a committee to co-operate with the officer in charge of this institution "in enriching its stores of dental literature and museum specimens."

Your Committee assumed the duties imposed, conscious of its own insufficiency for a work of such magnitude and vital importance. As we have labored to foster a loyal support of this cause and to promote an active effort for its advancement, we have become greatly impressed, as any one must who gives special attention to the matter, with the immense value of the opportunity herein afforded the dental profession to accomplish essential objects otherwise impossible of attainment. Never was there opportunity more freely offered a profession to demonstrate its value, to acquire a higher rank among the learned callings, to acquaint the professions and the general public with its achievements, and to secure the preservation, classification, exhibition and facilities for the study of all things pertaining to it of present or future historical and educational value. It would be with great loss of prestige and altogether inconsistent with the general course and progressive spirit of the dental profession if we fail to utilize equally with other specialists, and the general physicians and surgeons of the country, the immeasurable advantages of this institution, covering as it does, within a building erected and maintained by the Government for the purpose, all available matters pertaining to every branch of medicine and surgery.

The Museum, in several respects the rarest of its kind extant, contains more than 35,000 specimens, and, like the Library, is open to the public—the intellectual property of all professions and classes.

Its dental section may be made its most attractive department and the greatest object-lesson of its kind in the world, if the efforts of the management are met with a corresponding interest on the part of dentists. Dr. D. L. Huntington, Deputy Surgeon General, the chief officer of this institution, to whom this Committee is indebted for especial courtesy, has shown the most gratifying interest and encouraging effort in the development of the dental section. He has recently acquired by purchase a number of valuable and beautifully mounted specimens, most of which are rare. He also proposes to transfer to the dental section such objects as are of special interest to dentists which are now classified in other sections or distributed through the large general collection. This will enrich our section with specimens illustrating the effect of various diseases on the maxillary bones and the teeth, and with many other valuable objects which dentists could never acquire from their own resources.

The number of accessions, so far, directly from the efforts of this Committee is small—perhaps, one hundred.

We are pleased, however, to report such evidence of interest on the part of individuals with whom we have corresponded as warrant the expectation of contributions of the kind especially needed, namely: series of models, apparatus, drawings, etc., illustrating various operations, methods of treatment and their results. We also hope the leading college faculties will fully illustrate their methods of training, and otherwise utilize this institution to impart a knowledge of the extent and character of college studies and of the nature and import of the subjects taught. We are also greatly encouraged by the fact that quite a number of State and local societies have given heed to the action of the A. D. A., several formally endorsing its action in the premise and appointing committees auxiliary to this Committee. The societies so acting are the State societies of North Carolina, South Carolina, Maryland, District of Columbia, New Jersey, Pennsylvania, Tennessee and Mississippi, and the Valley Dental Society, a section of the Massachusetts State Society. From this, large visible results are certain to follow soon.

It is impossible to detail the kind of specimens desired. It is safe to say, however, that anything illustrative of any part of the subject of dentistry, or which would, alone or in connection with other specimens, throw light on the etiology, pathology, or treatment of the diseases and deformities of the teeth, jaws, etc., would attain a greatly-enhanced value by being placed here as parts of a complete collection.

The Army Medical Library is, admittedly throughout the world, the largest and most complete of its kind in existence. It contains three-fourths of the medical literature of the world and nine-tenths of the medical literature of recent years. There is a constant daily addition to its 120,000 bound volumes, 200,000 pamphlets and 1,200 current periodicals. Its literature is not only greater in volume than the medical literature of either the Library of the British Museum or the National Library of France, but covers a wider field and forms a better practical reference and working collection. The Library has acquired by purchase a large and choice collection of literature in English and other languages relating to dentistry. The voluntary *contributions of publishers and authors* would permit the money available for the purchase of their works to be used in other directions equally as essential to the purposes of the institution.

It would be impossible to exemplify the utilitarian purposes of such an institution as the Army Medical Museum and Library, or to say in how many ways such a great depository may be made available for the acquirement and dissemination of knowledge. Everything placed there receives and imparts light, and is enhanced in value by association for purposes of contrast and comparison. It affords the only legitimate means of reaching and teaching its many thousands of intelligent visitors and advanced students, who come from the various professions and better classes everywhere to return and impart to others the information acquired.

We commend this interest as worthy the sincere, constant and active support of every member of the dental profession, and declare the broad plans of the institution, the liberal spirit of its officers and the generous appropriations of Congress for its maintenance, ample, with the co operation of the dental profession, for the purpose of placing dentistry on a higher plane and consummating a work of immeasurable historical and educational value.

We respectfully recommend as a feasible plan of continuing the line of work commenced by this Committee; first, the appointment of five members of this Association as a National Committee charged with the duty of promoting the effort to build up a great National Dental Museum and Library; second, that this Association recommend the appointment of committees auxiliary to this National Committee by each of the local, State and other Dental Societies in the United States; third, that the sum of One

Hundred Dollars be appropriated, to be used with other donations, for the purpose of defraying necessary expenses of the National Committee.

Respectfully submitted,

WMS. DONNALLY, *Chairman.*
H. J. MCKELLOPS.
HENRY W. MORGAN.
J. TAFT.

[Presented to, and adopted by, the American Dental Association August 4th, 1896.]

---

### CHICAGO DENTAL SOCIETY.

AMERICAN JOURNAL OF DENTAL SCIENCE :

The Chicago Dental Society will celebrate its 33rd Anniversary, Monday and Tuesday, February 1st and 2nd, 1897, by giving a clinic with about twenty-five operators, each merning from 9 to 12 a. m. Papers will be read Monday afternoon and evening and Tuesday afternoon, closing the exercises with a dinner at 6.30 p. m. Members of the profession are cordially invited to be present. Headquarters for visitors will be at the Palmer House where special rates may be obtained.

This will be the first attempt on the part of the Chicago dentists to entertain their friends since the World's Fair, and they hope to have a large attendance.

Full programmes will be issued about January 15th, giving the location of clinic rooms, etc.

LOUIS OTTOFY. President.

A. H. PECK, Secretary.

Clinic Committee :
E. D. SWAIN,
J. W. WASSALL,
LOUIS OTTOFY,
D. M. CATTELL,
A. W. HARLAN, Chairman.
1000 Masonic Temple.

### ODONTOGRAPHIC SOCIETY OF CHICAGO.

At the annual meeting of the Odontographic Society of Chicago held Dec. 14th, 1896. The election of officers resulted as follows: President, Geo. B. Perry; Vice-President, G. W. Schwartz; Treasurer, Edmund Noyes; Secretary, H. H. Wilson; Member of Board of Directors, W. H. Fox; Board of Censors, E. K. Bennington, Chairman; A. G. Johnson and H. J. Goslee.

H. H. WILSON, Secretary.

### To Readers and Correspondents.

Communications intended for publication, and exchange journals and papers, should be addressed to the Editor of The American Journal of Dental Science, No. 845 N. Eutaw St. Baltimore, Md. All letters relating to the business of the Journal, or containing cash remittances, to be directed to Snowden & Cowman Mfg. Co., 9 W. Fayette Street, Baltimore, Md. Articles for publication should be received by the Editor at least two weeks previous to the first of the month on which the number in which it is designed for them to appear is issued.

☞ The American Journal of Dental Science will contain fifty pages of reading matter in each number, making a volume of about Six Hundred pages,

EXCLUSIVE OF ADVERTISEMENTS.

